# A Review of Equine Sleep: Implications for Equine Welfare

**DOI:** 10.3389/fvets.2022.916737

**Published:** 2022-08-17

**Authors:** Linda Greening, Sebastian McBride

**Affiliations:** ^1^Hartpury University and Hartpury College, Gloucester, United Kingdom; ^2^Institute of Biological, Environmental and Rural Science, Aberystwyth University, Aberystwyth, United Kingdom

**Keywords:** equine, horse, behavior, sleep cycle, sleep quality, sleep quantity, sleep deprivation, welfare

## Abstract

Sleep is a significant biological requirement for all living mammals due to its restorative properties and its cognitive role in memory consolidation. Sleep is ubiquitous amongst all mammals but sleep profiles differ between species dependent upon a range of biological and environmental factors. Given the functional importance of sleep, it is important to understand these differences in order to ensure good physical and psychological wellbeing for domesticated animals. This review focuses specifically on the domestic horse and aims to consolidate current information on equine sleep, in relation to other species, in order to (a) identify both quantitatively and qualitatively what constitutes normal sleep in the horse, (b) identify optimal methods to measure equine sleep (logistically and in terms of accuracy), (c) determine whether changes in equine sleep quantity and quality reflect changes in the animal's welfare, and (d) recognize the primary factors that affect the quantity and quality of equine sleep. The review then discusses gaps in current knowledge and uses this information to identify and set the direction of future equine sleep research with the ultimate aim of improving equine performance and welfare. The conclusions from this review are also contextualized within the current discussions around the “social license” of horse use from a welfare perspective.

## Introduction

The primary aim of this review is to create a greater understanding of equine sleep and to discuss and identify its role in equine welfare. Although a fundamental process in all mammals, sleep is not commonly considered as a factor that affects animal welfare (1). For example, in many of the animal welfare frameworks and guidelines, there are specific references to factors such as sufficient air and light, food and water, adequate spaces for movement and contact with conspecifics, but not to creating environments that facilitate maximum levels of species-specific sleep. In addition, whilst some standards have been written to ensure the provision of species-appropriate spaces to rest (e.g., Department for the Environment, Food and Rural Affairs, UK), very little information exists about how to implement this in practice. Furthermore, very little information exists that describes species-appropriate spaces to promote rest in the domestic environment. In this respect, more consideration needs to be given to understanding species-specific sleep requirements in a domestic setting and how best to accommodate these in order to help optimize animal welfare.

Although this review focuses primarily on the horse, many of the concepts within the review are applicable to other domestic animal species and thus some of the conclusions drawn are potentially generalizable to other species held within domestic, captive, farm or laboratory environments. To understand the role of sleep in animal welfare requires an understanding of the normal sleep states and sleep profile for the species in question, as well as how those states and profiles can be measured. The review, therefore, also provides a comparative and evolutionary assessment of equine sleep to create a detailed sense of the normal equine sleep profile, as well as the basic sleep requirements of the horse. The different possible approaches to measuring equine sleep for applied purposes are also discussed, followed by a review of what is currently known about factors affecting equine sleep.

## Definition, Sleep Stages and Variation of Animal Sleep

### Definition and Different Sleep Stages

Sleep is defined as a maintained state of quiescence characterized by relative inactivity, loss of consciousness (2) and/or increased thresholds of arousal to environmental stimuli (3, 4). Individuals tend to adopt a distinct and sustained species-typical posture during sleep usually in a specific or preferred location (3). Sleep is also characterized as a rapidly reversible state when compared to other similar physiological states such as hibernation and torpor (3, 5, 6). Two main processes regulate the occurrence of sleep; circadian rhythms organize the timing of sleep during the 24-h cycle, whilst homeostatic mechanisms determine the amount of sleep that a species requires (7–9).

Electroencephalogram (EEG) profiles have identified two primary states of sleep for a range of species, non-rapid eye movement (NREM) and rapid eye movement (REM) sleep. NREM, also known as slow wave sleep (SWS), has been further divided into four stages (N1–N4, described in detail under the section Comparison of Human and Equine Sleep Stages). The transition into and out of sleep is characterized by drowsiness or quiet wakefulness and sleep onset is described as a gradual process until the first occurrence of NREM N2 sleep (10). During a normal sleep episode, individuals cycle between bouts of NREM and REM sleep. Individuals usually engage in behavioral rituals prior to sleep (e.g., circling the nest/yawning etc.) (11) however the process of falling asleep is often irregular between individuals (10).

There are a number of specific behavioral and physiological correlates of NREM and REM sleep. For example, the body loses muscle tone and suspends central homeostasis during REM sleep (REM atonia), resulting in fluctuations in the autonomic nervous system (12). N1 and N2 NREM sleep are often associated with slow eye movements and low arousal thresholds which then reduce and increase respectively during the transition to N3 and N4 NREM sleep stages (see Section Enhancing the Behavioral Measurement of Equine Sleep Quantity). Whilst this profile is common amongst the majority of mammals, there are exceptions for example, in the monotremes, features of both REM and NREM sleep are merged into a single sleep-like state (13) or REM sleep activity is only found in the brainstem region of the brain (14).

### Micro-Arousals, Wake Sequences and Wakefulness

Sleep stages are an ever-changing dynamic process due in part to the cyclic nature of sleep and the occurrence of arousing stimuli in the internal and external environment. Stages of sleep are, thus, often interrupted by either micro-arousals or slightly longer wake sequences and can be broken completely into a full state of wakefulness. During sleep, the brain continues to interpret information such that arousal eliciting factors work against sleep promoting forces. When there is higher pressure to sleep during the descending loop of the sleep cycle (N1–N4), phasic changes from sensory input often don't disrupt sleep and can in fact result in deepening of SWS (15). During the ascending loop of the cycle (N4 to REM), however, when there is lower sleep pressure, sensory input has a more pronounced modifying and disrupting influence on sleep. These phasic changes are referred to as micro-arousals (MA), defined as momentary adaptations to vigilance levels in response to internal and external sensory input. Within the EEG profile, MA are seconds in duration and are associated with the emergence of K-complexes (high and low voltage waveform) (15). According to the American Sleep Disorder Association, a micro-arousal should be scored when there is an increase in EEG frequency for 3 s or more but that K-complexes should not be scored as arousals unless accompanied by increased EEG frequencies (16).

Wake sequences describe longer periods of arousal when the animal is no longer within a NREM or REM state. The duration of these sequences is often species-dependent and can occur either within or at the end of a sleep cycle. For example, in rodents, brief wake sequences (<300 s) have been described interrupting periods of NREM or REM within the sleep cycle, whilst longer disturbances (>300 s) have been described between sleep cycles (17).

The definition of full wakefulness from sleep relates to the probability of re-entering sleep within a specified time period. There are a number of specific neurophysiological events that occur on entering full wakefulness that are largely driven by the activation of efferent hypocretin neurons from the lateral hypothalamus (18). One of the main differences between sleep and wakefulness is increased sympathetic tone and decreased parasympathetic tone that maintains most organ systems in a state of action or readiness. In humans, spontaneous awakenings lasting longer than 3 mins are generally acknowledged as a state of wakefulness that modifies the sleep cycle (19).

### Ecological and Biological Factors Affecting Mammalian Sleep

A comparison of average total sleep time between polyphasic mammalian species reveals large-scale differences and several factors have been proposed to explain this (Table 1). One of the primary factors is body mass which negatively correlates with total sleep time (22, 23). This is considered to reflect the risk of predation for larger prey species which, due to their size, are required to sleep in exposed locations (24), and thus tend to not enter into prolonged periods of deeper stages of NREM sleep. The severity of predation and safety of sleeping place are often scored from one to five using a sleep exposure index; a score of one is given to a sleep site that is well-protected with minimum predation, a score of five is a sleep site of high predator risk/exposure (20, 22) (Table 1). Mammalian prey species sleeping in riskier locations are commonly observed to engage in lower proportions of REM sleep, as this sleep stage can only be attained in the recumbent position (20).

**Table 1:** Comparison between sleep profiles and biological characteristics of various bi-hemispheric species, ordered by high to low sleep exposure indices (3, 20, 21).

**Species**	**NREM (h/day)**	**REM (h/day)**	**Total sleep (h/day)**	**Brain mass (g)**	**Ave. body mass (g)**	**Ave. proportion brain/body mass**	**BMR (cm^**3**^ O_**2**_ h^**−1**^)**	**Gestation period (days)**	Sleep exposure index^*^
Horse (*Equus caballus*)	2.98	0.67	3.85	534.0	260,000	0.21%	65,000.0	337.0	5
Cow (*Bos taurus*)	3.2	0.8	4.0	460.0	272,000	0.17%	46,240.0	280.7	5
Sheep (*Ovis aries*)	3.3	0.6	3.8	100.0	30,000	0.33%	10,200.0	146.3	5
Goat (*Capra aegagrus*)	4.7	0.7	3.8	115.0	29,000	0.4%	6,840.0	163.9	5
Pig (*Sus scrofa*)	6.4	1.9	8.4	180.0	75,000	0.24%	8,250.0	117.0	4
Dog (*Canis familiaris*)	7.1	1.6	10.7	70.0	14,000	0.52%	–	62.0	2
Cat (*Felis silvestris*)	10.0	3.2	13.2	28.4	3,260	0.87%	2,314.6	63.9	1.5
House mouse (*Mus musculus*)	11.9	1.3	12.8	0.4	21	1.90%	69.7	21.2	1.33
Human (*Homo sapiens*)	6.1	1.9	8.0	1,320.0	62,000	2.13%	14,700.0	280.1	1

* *Sleep exposure index concerns a measure of predation risk based on vulnerability associated with sleep site. The index ranks relative exposure of a given species at its typical sleep quarters in the wild, where 1 = low risk e.g., caves/burrows and 7 = high risk, e.g., open water (22)*.

Another factor influencing sleep time is the degree of species encephalization which positively correlates with total REM sleep and supports the role of REM in memory consolidation (22). However, this is a complex relationship due to additional influencing factors including whether the species bears precocial or altricial young (21). For example, precocial species that experience longer gestation periods have high levels of brain maturity at birth so that offspring can adapt quickly to the external world (25). This would suggest that precocial species would have higher levels of REM sleep, however, because these animals tend to live in exposed environments of high predation risk, the total level of REM sleep tends to be lower (23). In contrast, neonates of less developed altricial species display larger amounts of REM sleep (7) potentially as the result of being maintained in a protected safer environment (21). Research has also shown a negative correlation between total sleep time and basal metabolic rate (BMR) with the hypothesis being that species with greater energy expenditure relative to their size are required to make a trade-off between sleep and foraging, with foraging superseding sleep (7, 26). Additionally, higher BMR is linked to higher levels of restorative neurophysiological processes thus potentially reducing the need for long periods of restorative sleep (22).

Similar to most mammalian species, the horse engages in stages of wakefulness, rapid eye movement (REM) sleep, and non-rapid eye movement (NREM) sleep. Periods of drowsiness or light sleep have been recorded before the horse experiences NREM/REM (27). In comparison to the mammals listed in Table 1, the horse is one of the species engaging in the least amount of sleep (equal rank 1 with sheep and goat). This fits with its ecological and biological characteristics; a precocial species with a high gestation period (rank 1), high basal metabolic rate (rank 1) and high body mass (rank 2) and a sleep exposure index of five (equal rank 1 with cow, sheep and goat).

Additional to the factors in the table, which from an evolutionary perspective have determined animal sleep duration and patterns, is the social context in which sleep is performed. Protection during sleep can be provided through group living in social species (28), for example, in the equine herd, individuals are often observed to interchange between recumbent and standing positions as a supposed rota system of group vigilance (29). These innate behavioral characteristics are observed in both free-living horses (those populations receiving very little human management), and domesticated horses (those born into an artificial environment) (29). This element of protection however, is not considered to increase sleep quotas overall where often, for large herbivorous species, the continued trade-off between risk of predation and need to forage maintains a comparatively low total sleep time (30). This form of synchronized social sleep may, however, still be critical to vigilance levels and thus the level of micro-arousals and wake sequences the animal experiences during sleep. In the domestic environment, the social context as well as several other factors (e.g., athletic training, nutrition, housing environment) can be substantially different to the free-living environment. These factors will therefore have an impact on sleep patterns in the horse and are discussed in more detail under the section Factors Reducing Sleep.

### Comparison of Human and Equine Sleep Stages

In the awake state, human EEG patterns present as two types of waves, beta and alpha. Beta waves are associated with full wakefulness and have the highest frequency (13–30 Hz) and lowest amplitude (10–30 μV) (31). Beta wave forms have few rhythmic components and are thus more dyssynchronous than other wave forms (32). Beta wave forms have been measured in the horse during wakefulness and active states (33). During more inactive states of reported relaxation in humans, alpha brain waves predominate (34). These brain waves are slower (8–13 Hz) with increased amplitude and are more synchronous in nature (35) with occasional bursts of high frequency (beta) wave forms associated with periodic alertness (34). Alpha waves have been measured in the horse (33, 36) and are associated with the behavioral markers of weight bearing on 2 fore and 1 hind limb with the head above withers (36). The transition into the sleep state is associated with a further reduction in brain wave frequency to a theta wave state (3–7 Hz) but with occasional presentation of alpha waves (8–13 Hz) (37). This is referred to as the N1 stage of NREM sleep and is often preceded by a state of drowsiness, particularly in animals (38). Full sleep onset is typically considered to occur at the N2 stage of NREM, here the EEG profile is similar to N1 (theta waves, 3–7 Hz) but now also contains sleep spindles and/or K complexes with at least 1 sleep spindle or K-complex occurring per 30 s on a N1 background (10). In humans, the N2 sleep stage tends to predominate during periods of sleep across all ages (39) and this sleep stage is routinely observed in the horse (36, 39). The next two stages of NREM sleep (N3–N4) are collectively referred to as SWS and are characterized by slow wave oscillations (0.5–2.0 Hz) of high amplitude (75 mv), referred to as delta waves. The N3 stage of SWS is defined as having between 20 and 50% delta waves whereas the N4 stage contains above 50% (40). More recently within the human literature the N3 and N4 stages of sleep have been merged (41). N3 but not N4 NREM sleep has been explicitly reported in horses and is commonly referred to as SWS (36, 42).

REM sleep is often referred to as “paradoxical” sleep because of the mixed frequency (3–30 Hz), low amplitude (10–30 uV) nature of brain waves associated with this sleep stage that is often also observed during wakefulness (40). REM sleep is associated with tonic suppression of skeletal muscle tone and reflexes through inhibition of the brain stem and spinal motor neurons (43) but with episodic bursts of rapid eye movements and muscle twitches arising from ponto-geniculo-occipital brain waves (37). REM sleep has been observed in the horse and is associated predominantly with lateral recumbency (reflecting full muscle atonia) (42) and also sternal recumbency with the head propped on the floor (36). In some instances, short episodes of REM sleep occur in the horse whilst standing accompanied with complete loss of tone in the neck muscles, head dropping to just above the floor and the horse buckling forelimbs with the muzzle hitting the floor (36). REM sleep in the horse is also associated with rapid eye movements and rhythmic ear twitching (36). In order to characterize and compare sleep profiles within and between species, standard descriptive terminology of the different sleep states in accordance with previous definitions is presented in Table 2, alongside some additional terminology that may be particular to the equine sleep profile.

**Table 2:** A glossary of terms and standard definitions of sleep states (19, 44, 45) with visualization of specific terms using an example equine hypnogram.

**Term**	**Definition**
Sleep onset	An episode of sleep lasting >1 minute and containing at least 1 min of sleep other than N1 NREM (19).
Sleep cycle	The interval from sleep onset (see definition above) to the start of a period of wakefulness that is greater than 3 mins (19), containing sequences of REM, NREM and wakefulness.
Epoch	A short interval of arbitrarily defined length (usually 20–60 s) of sleep stage normally determined from a polygraphic sleep recording (44).
Micro-arousal	A sudden transient elevation of the vigilance level due to arousal stimuli or to spontaneous vigilance level oscillations incorporating low-voltage fast-rhythm electroencephalographic (EEG) arousals and high-amplitude EEG bursts (46)
Sequence	A consecutive series of epochs in the same sleep stage (44) e.g. A REM sequence is a series of consecutive epochs of REM sleep uninterrupted by any other sleep stage or state
Episode	A series of consecutive sequences of the same stage of sleep or the same state which may be interrupted for a short time by another sleep stage or state (44) e.g., REM episode is a series of consecutive sequences of REM sleep which are separated by less than 15 mins of NREM sleep or 3 mins of wakefulness
Sleep episode (duration)	Portion of the sleep-wake cycle from sleep onset to last epoch of sleep, which may include sequences of wakefulness (measured by the number of minutes from sleep onset to the end of the last sleep epoch)
REM-NREM cycle (length)	A general term used to describe cyclic alteration between REM and/or NREM sleep measured in units of time which must be clearly defined e.g., “the end of one REM episode to the end of the next REM episode” and whether the cycle analyzed began with REM or NREM.
Somnolence	A state of desire for sleeping/being drowsy/ready to fall asleep.
Torpor	A state of decreased physiological activity usually involving reduced body temperature and metabolic rate that enables the animal to survive periods of reduced food availability.
Uni-hemispheric	Sleep is induced in only one cerebral hemisphere whilst the other remains awake, resulting in asymmetric eye closure and sleeping postures (45).
Bi-hemispheric	Sleep involves both cerebral hemispheres, characterized by closure of both eyes and symmetric body muscular hypertonia or atonia.
Monophasic	Sleep occurs in one long period, usually during the night
Polyphasic	Episodes of sleep that occur during the day and/or night
Polysomnography	Multiple physiological measurements taken to measure sleep including electroencephalography, electrooculography, electromyography, electrocardiography, breathing frequency and body temperature.
Zeitgeber	A rhythmically occurring natural phenomenon which acts as a cue in the regulation of the body's circadian rhythms.
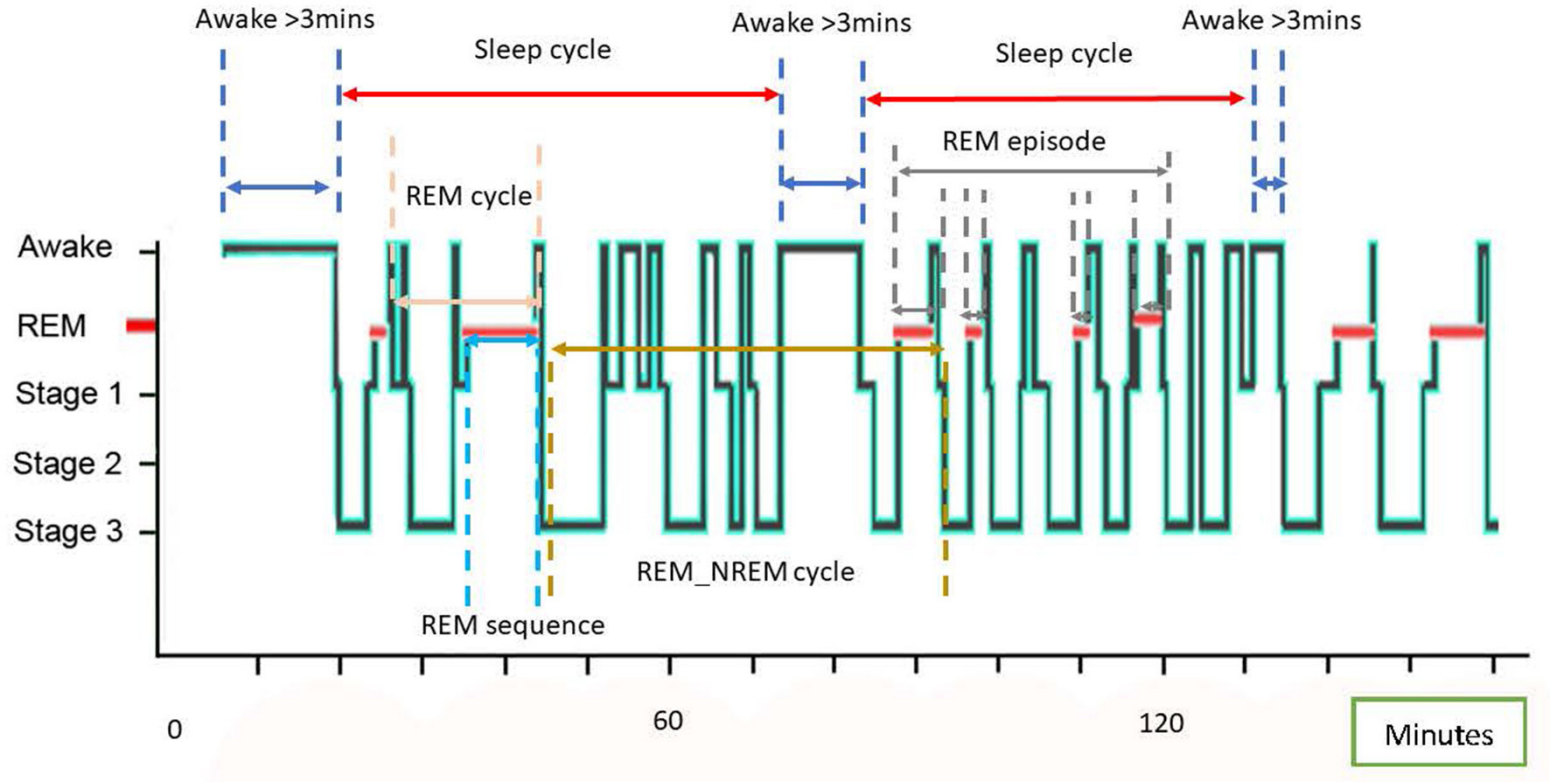	

### Comparisons of the Human and Equine Sleep Cycle

#### General Characteristics of the Human Sleep Cycle

**Figure 1:**
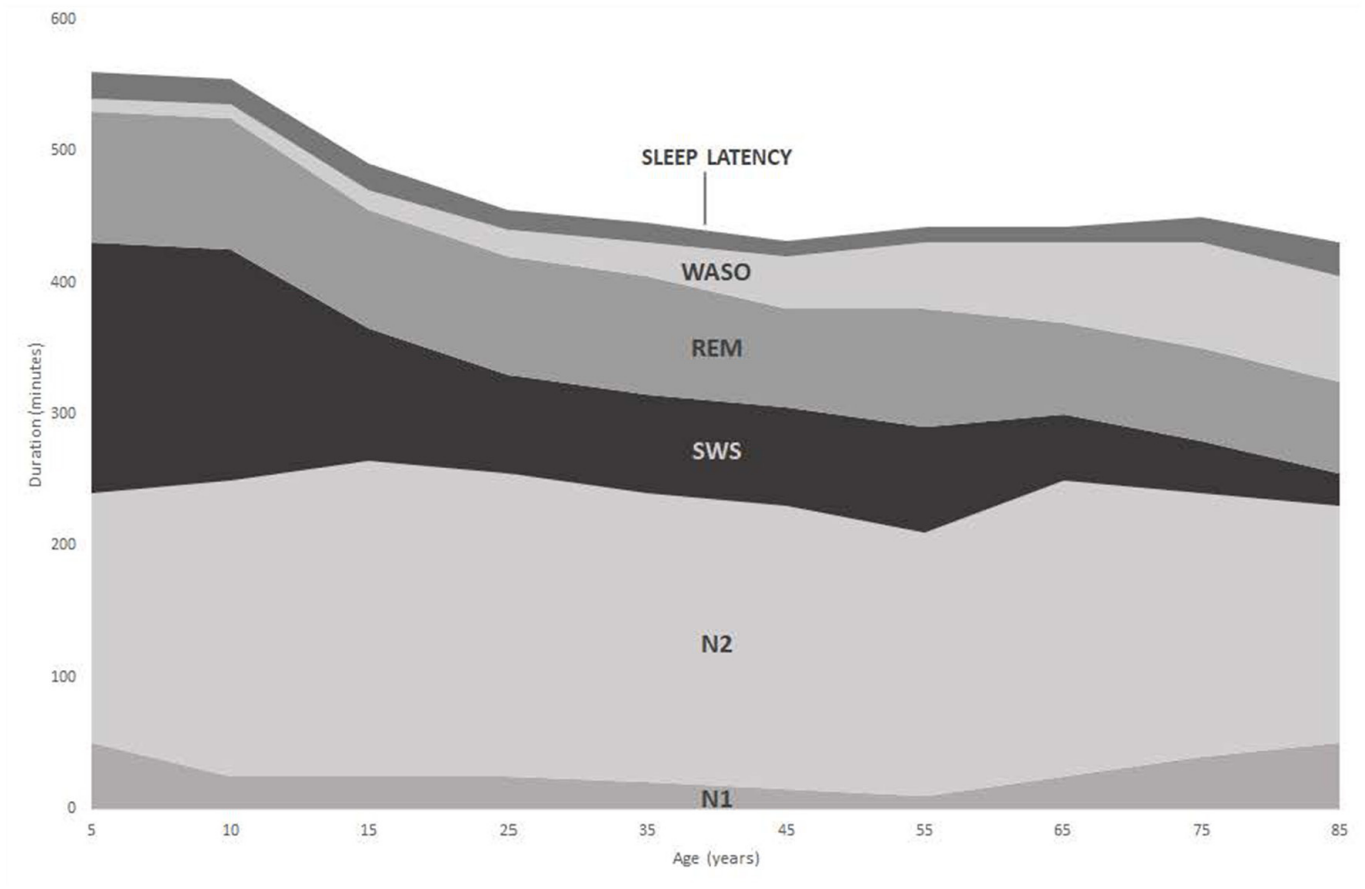
Changes in duration of sleep stages with increasing age [adapted from Ohayon et al. (50)].

**Figure 2:**
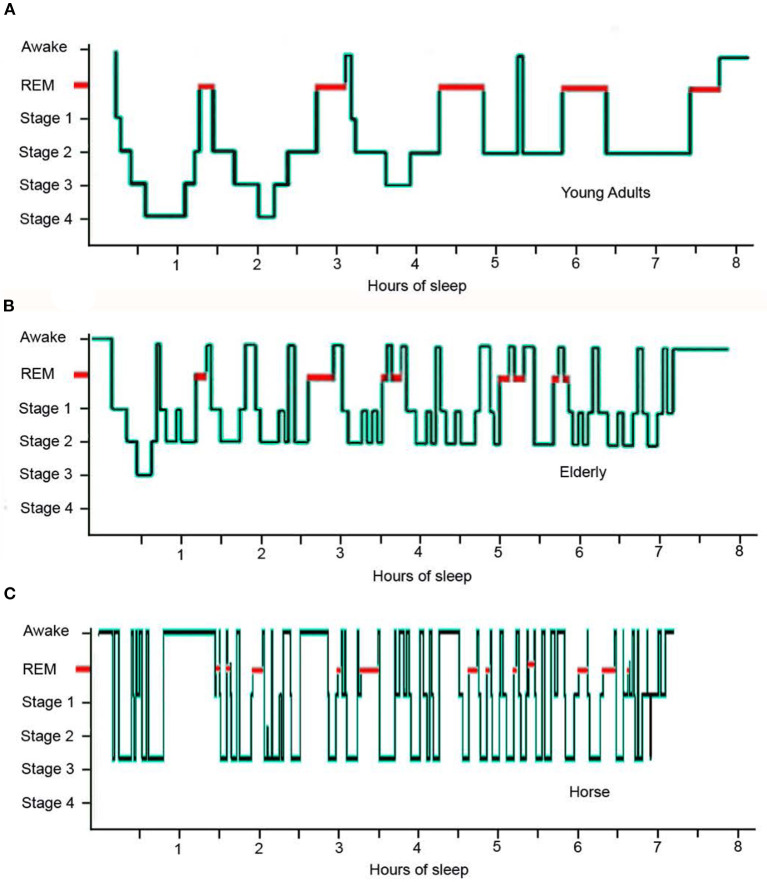
Hypnogram comparing sleep architecture of a typical young human adult, an elderly human adult and an adult horse over an eight hour sleep episode: **(A)** the young adult experiences 5 REM sequences and 2 short sequences of wakefulness; **(B)** the elderly individual experiences 8 disturbed REM sequences and multiple sequences of wakefulness accompanied by a distinct lack of stage 4 sleep; **(C)** the horse experiences 13 disturbed REM sequences and multiple sequences of wakefulness accompanied by a distinct lack of stage 4 sleep [adapted from (42, 69)].

The human sleep cycle involves predictable patterns of sleep sequences and is commonly reported to last on average 90–100 mins, measured from the end of one REM sequence to the end of the next (47). After initial sleep onset (beyond the N1 stage), normal progression through a sleep episode follows N2 into SWS (N3–N4), followed by either N1 and/or REM. On average, 20–25% of the total sleep time (TST) is occupied by REM sleep occurring in four to six sequences (47). At the end of the first REM sequence, SWS re-emerges within subsequent cycles of sleep. As the overall episode of sleep progresses however, SWS sequences diminish in duration and are superseded by more time within N1 and N2 stages (47, 48). In this respect, sleep cycles earlier during the night are shorter (on average 70–90 mins) than later cycles which can last between 90 and 100 mins (49). Across a complete sleep episode, N1 is reported to account for 2–5% of TST, N2 accounts for ~45–55% TST, whilst N3 constitutes 5–15% TST although this varies with age (Figures 1, 2).

A normal pattern of human sleep is often difficult to characterize due to individual sleep duration variability, compounded by factors such as duration of prior waking, staying up late, and waking by alarm (37). In addition, internal (e.g., drugs) and external (e.g., sound) stimuli will also influence the dynamic process of sleep (15). Sleep architecture also changes considerably with age. Twelve month old infants typically sleep for 14–15 h per day (52), which drops to 11 h by age 5, whilst adolescents require 9–10 h of sleep each night (53). Differences in the profile of sleep stages within a sleep episode are also apparent between young adults and the elderly (Figure 2). This is especially true when comparing the increasing number of brief arousals and wake sequence as a function of age (49, 54). In total, the amount of time awake after initial sleep onset (WASO) increases with increasing age (Figures 1, 2) along with a decrease in duration of REM and SWS sleep. Since arousal thresholds are higher during SWS and this sleep stage decreases with age, older adults become prone to experience more frequent awakening during a sleep episode (53).

#### Characteristics of the Equine Sleep Cycle in Comparison With the Human Sleep Cycle

Most mammalian species, including the horse, are polyphasic sleepers engaging in multiple sleep episodes across 24 h (7). This is considered to be an adaptation for increased vigilance (and thus survival) but may also indicate limited ability to sustain wakefulness (55). Table 3 presents data on all reported studies of equine sleep to date. This table sets out a number of sleep parameters to help define the characteristics of equine sleep and the equine sleep cycle, and allow for comparison with the human equivalent sleep profile.

**Table 3:** Reported measurements and characteristics of the equine and human sleep profile.

**Study**	**Number of individuals**	**Mean age (age range)**	**Hours recorded (from/to)**	**Mean total sleep time**	**Mean total NREM duration**	**Mean total REM/lateral recumbency duration**	Mean duration of NREM sequences^**^(mean No. Of NREM sequences/TST)	Mean duration of REM sequences^**^(mean No. Of REM sequences/TST)	Mean duration of NREM episode^**^(mean no.)	Mean duration of sleep cycle^**^[mean no. (range)]	Mean number of wake sequences^**^(<3 min) within a sleep cycle	Mean duration of wake sequences^**^with in a sleep cycle
Wohr et al. (56)	7	Adult horses	7 h at night	210.0 min 50% TOT	40.0 min 65% TST 9.52% TOT	30.0 min 5%TST 7.14% TOT	NM	NM	NM	NM	NM	NM
Greening et al. (57)	10	14.9 years	24 h	311.8 min 21.7% TOT	236.4 min 76.1% TST	104.0 min 23.9% TST	NM	NM	NM	NM	NM	NM
Chung et al. (58)	15	Adult horses	24 h (8 am−8 am)	65.0 min 4.5% TOT	57.0 min excl stand sleep 88% TST 4% TOT	8.0 min 12% TST 0.5% TOT	NM	NM	NM	NM	NM	NM
Dallaire and Ruckebusch (59)	5	(6 months to 6 years)	12 h (8 am−8 am)	199.5 min 27.2% TOT	151.5 min 75.9% TST 21% TOT	48.0 min 24.1% TST 6.7% TOT	6.48 min	4.0 min	NM	40.78 min (5.5)	NM	NM
Dallaire and Ruckebusch, (60)	3	(6 months to 6 years)	12 h (18.30 pm−6.30 am)	189.3 min 26.3% TOT	145.7 min 77% TST 20.3% TOT	43.6 min 23% TST 6.1% TOT	NM	NM	NM	NM	NM	NM
Greening et al. (61)	10	7.3 years	12 h (7 pm−7 am)	382.0 min 53% TOT	355.0 min 93% TST 49% TOT	27.0 min 7% TST 4% TOT	NM	NM	NM	NM	NM	NM
Hartman and Greening (62)	7	11.7 years (6–16 years)	10 h (8.30 pm−6.30 am)	299.0 min 49.8% TOT	265.0 min 88.7% TST 44.1% TOT	33.8 min (scans at 2 min intervals) 11.3% TST 5.6% TOT	NM	NM	NM	NM	NM	NM
Kalus (42)	7	14.1 years (8–20 years)	7 h (10.30 pm−5.30 am)	203.0 min 51% TOT 203/420 = 48.3%	131.1 min 65.5% TST 131.1/203 = 64.6%	31.3 min 15.5% TST 31.3/203 = 15.4%	5.22 min (25.88)	2.38 min (7.11)	17.14 min (3.04)	40.7 min (2.63)	6.89	0.96 min
Kwiatkowska-Stenzel et al. (63)	8	(4–13 years)	48 h (x3 12.30 pm−4.30 am)	321.3 min 33.5% TOT	265.3 min 82.6% TST 27.6% TOT	56.0 min 17.4% TST 5.8% TOT	NM	NM	NM	NM	NM	NM
Williams et al. (36)	6	(4–13 years)	^*^12 h (8 am−8 am)	166.4 min	158.2 min (95.1% TST)	8.16 (4.9%TST)	3.37 min (52)	0.91 min (14.6)	NM	NM	NM	NM
Ruckebusch (64)	4	4 years	At night	218.0 min	181.0 min 83% TST	37.0 min 17% TST	9.0 min (18)	4.8 min	NM	NM	NM	NM
Ruckebusch et al. (65)	2	Unknown	12 h (8 pm−8 am)	262.1 min 36.4% TOT	212.42 min 81% TST 29.5% TOT	49.7 min 19% TST 6.9% TOT	NM	5.02 min (11.3)	NM	NM	NM	NM
Ruckebusch, (27)	3	Adult horses	10 h (over-night)	172.0 min 28.7% TOT	125.0 min 72.7% TST 20.8% TOT	47.0 min 27.3% TST 7.8% TOT	NM	5.22 min (9)	NM	NM	NM	NM
**Mean**	**7 horses**	**10.4 years**	**15.83 h**	**230.72 min (24.3% of average TOT)**	**178.74 min (77.5% of average TST)**	**40.27 min (17.5% of average TST)**	**6.02 min (31.96)**	**3.72 min** **(10.5)**	**17.14 mins**	**40.74 min** **[4.1 (1–6)]**	**6.89**	**0.96 min**
**SD**	**3.57**	**4.64**	**1.41**	**82.87**	**87.53**	**24.15**	**4.41**	**1.72**	**NA**	**0.06**	**NA**	**NA**
Human [Le Bon (66)]	78	27.8 (5–45 years)	7 h 50 min	433.26 min 91.6% TOT	350.72 min 74.1% TOT 80.9% TST	73.84 min 15.6% TOT 17.04% TST	NM	NM	101.36 mins	119.7 mins [4.23 (2–6)]	NM	NM
Human—children [Feinberg (67)]	21	13.8 (11.8–16.2 yrs)	NM	NM	NM	NM	77.5 min	22.7 min	NM	NM	*0.25*	1.64 min
Human—adult [Feinberg (67)]	13	31.5 (26.2–43.3 years)	NM	NM	NM	NM	62.0 min	24.0 min	NM	NM	0.77	2.75 min
Human—aged [Feinberg (67)]	9	77.3 (67.4–95.8 years)	NM	NM	NM	NM	64.4 min	20.3 min	NM	NM	0.9	11.56 min
Human—old [Carskadon et al. (68)]	24	(63–86 years)	48 h (10 pm−8 am)	426 min	355 min 83.33% TST	72 min 16.9% TST	NM	NM	NM	NM	NM	3.1 min
**Mean**	**29 people**	**37.6 years**	**48 h**	**429.63 min** **25.6% TOT**	**352.86 min 82.13% TST**	**72.92 min** **16.97% TST**	**67.97 min**	**22.33 min**	**101.36 mins**	**119.7 mins** **[4.23 (2–6)]**	**0.64**	**4.76 min**
**SD**	**28.04**	**27.54**	**NA**	**5.13**	**3.03**	**1.30**	**8.34**	**1.88**	**NA**	**NA**	**0.34**	**4.57**

* *Recording stated as “night”*.

** ***Episode** = A series of consecutive sequences of the same stage of sleep or the same state which may be interrupted for a short time by another sleep stage or state e.g., REM episode is a series of consecutive sequences of REM sleep which are separated by <15 min of NREM sleep or 3 min of wakefulness; **Sequence** = A consecutive series of epochs in the same sleep stage, e.g., a REM sequence is a series of consecutive epochs of REM sleep; **Sleep cycle** = The interval from sleep onset to the start of a period of wakefulness that is >3 min, containing sequences of REM, NREM and wakefulness, TOT total observation time, TST, total sleep time, NM, no measurement*.

##### Total Sleep Time

Studies of equine sleep describe on average 230.72 min (±82.87) total sleep time (TST). This is in comparison to an average TST of 429.63 min (±25.6) observed for humans (66, 68). Studies using EEG or ECoG reported mean total equine sleep duration ranging from 172 to 262 mins. Meanwhile, studies using behavioral observations record total equine sleep as between 65 and 382 min, depending on whether standing sleep was included and how total sleep time was defined. Differences in age of horses between studies (from 6 months to 20 years), use of sampling techniques, and total sleep observation time (e.g., 12 vs. 24 h) are also likely to cause variation within reported TST between studies. This latter point identifies the importance of using standardized methodology when taking a behavioral approach to quantifying animal sleep (discussed in more detail in Section Enhancing the Behavioral Measurement of Equine Sleep Quantity).

##### Total REM and Total NREM

For horses, NREM sleep consistently accounts for the largest proportion of total sleep time (77.50%) (mean 178.74 ± 87.53 min) compared to REM sleep (17.50% of total sleep time) (40.27 ± 24.15 min). Although the average total NREM and REM sleep duration is greater in humans (352.86 and 72.92 min respectively), the ratio of NREM to REM for human sleep (82 and 17%) is similar to the horse (66, 68), with a larger proportion of total sleep time devoted to NREM sleep for both species. In total, NREM and REM sleep constitute 95% of the total sleep time for the horse compared to 99% for the human which may be indicative of greater levels of wakefulness in the horse during sleep cycles.

##### Sleep Cycle Duration and REM and NREM Components of the Sleep Cycle (Sequences and Episodes)

The average number of sleep cycles for human sleep has been recorded at 4.23 with a range of 2–6 cycles per night that last for an average of 119.70 mins, ranging from 80.7 to 199.1 mins (66). The horse also engages in 2–6 cycles (averaging 4.1 cycles) but these are shorter on average than in humans (40.74 mins). Many equine studies only measure sleep at night and thus additional sleep cycles, that are known to occur during the day for the horse, will not have been taken into account. In this respect, the average value of 4.1 cycles pertains only to the nocturnal sleep profile.

In the horse, the average duration of NREM sequence within the total sleep time is 6.02 min (ranging from 3.37 to 9 min) with the average number of sequences being 31.96 (ranging from 18 to 52). This is substantially different to humans where the average duration of NREM sequence is 67.97 mins (ranging from 62 to 77.5 min) within the total sleep time. The number of these prolonged NREM sequences in humans during the total sleep time is therefore also substantially lower, with one sequence per sleep cycle and an average of 4 sleep cycles per night (66). This is reflected in recorded durations of NREM episodes which are substantially greater in humans (101.36 min) compared to the horse (17.14 min) (42, 66).

The average duration of REM sequence in the horse is 3.72 min (ranging from 0.91 to 5.13 min) and the average number of REM sequences is 10.5 (7.11–14.6) during the total sleep time. This is compared to an average duration of 22.33 min for humans with the average number of REM sequences equating to the average number of sleep cycles per night (40). This difference in REM sequence duration and frequency illustrates the fragmented nature of equine vs. human sleep (Figure 2).

##### Wake Sequences (Duration and Number)

The mean number of wake sequences per sleep cycle is 0.25 for children, 0.77 for adults, 0.9 for elderly adults and 6.89 for the horse (Table 3). The mean duration of wake sequences for children is 1.64 vs. 2.75 mins for adults, 11.56 mins for elderly adults and 0.96 mins for the horse. In this respect, elderly humans are experiencing wake sequences greater than 3 mins which are thus more likely to be complete breaks in the sleep cycle with a new sleep cycle restarting after the wakefulness has occurred. Thus, whilst the number of wake sequences within a sleep cycle for the elderly adult has greater similarities to the horse compared to younger humans, the nature of the wake sequence appears to be quite different (Figure 2). The increased number of long duration wake sequences in the elderly human is considered to reflect non-functional age-related changes in the brain (70), whereas the large number of short duration wake sequences in the horse is considered to serve a much more functional role in maintaining vigilance levels against predators (51).

In conclusion, the horse sleeps for ~50% of human total sleep time for periods of nocturnal observation. Although the nocturnal sleep cycle frequency appears comparable between humans and horses, the duration and frequency of sleep stages (NREM and REM) within the equine sleep cycle is much shorter compared to the human. This discrepancy is due in part to the higher frequency of short duration wake sequences that occur within sleep cycles, and the extended periods of wakefulness that occur between the equine sleep cycle. In this sense, the conventional definition of a sleep cycle in human terms (progressive stages of NREM followed by REM) may be less applicable to the horse, particularly given that the horse can cycle through sleep onset to wakefulness and display only NREM sleep. In this sense, equine sleep demonstrates greater similarity to the elderly human sleep profile. The data from the table also does not completely capture the polyphasic nature of equine sleep as many of the studies only record or observe the horse overnight. More complete 24 h studies are still needed therefore to provide a complete picture of the equine sleep profile.

## Enhancing the Behavioral Measurement of Equine Sleep Quantity

As previously discussed (Section Definition, Sleep Stages and Variation of Animal Sleep) EEG profiles, often in combination with polysomnography (PSG), give an accurate quantification of specific sleep states in a range of species including the horse (3), however, equipment is needs to be specialized and the use of surface electrodes often produces data loss thus producing incomplete data sets (71). The behavioral quantification of sleep has historically provided a viable alternative to EEG measurement (3) and thus, there is an argument for developing this approach for the measurement of equine sleep when EEG equipment is not available. One of the primary problems with this approach, however, is that the horse is capable of achieving various stages of sleep in multiple body positions (Table 4). For example, NREM N1–N3 sleep stages are differentiated only by the position of the eye lid and the position of the poll in relation to the withers when the horse adopts these sleep states whilst standing (36). Although NREM sleep tends to occur in standing positions, it can also occur when the horse is recumbent making the differentiation between NREM and REM sleep states more difficult. REM sleep, due to muscle atonia, can only occur in recumbent states (lateral and sternal) with the muzzle being placed on the floor in the sternal position (56). Some horses (although rare) can enter a REM sleep state in a standing position but only momentarily before muscle atonia occurs and the horse collapses bringing it out of sleep (36).

**Table 4:** Behavioral markers of different EEG sleep states in the horse.

**Stage of sleep**	**Behavioral markers**								
	**Leg resting**	**Eye lid partially shut**	**Ears non-vertical**	**Eye lid completely shut**	**Poll below withers**	**Sternally recumbent**	**Muzzle on the floor**	**Laterally recumbent**	**Ears or eyes twitching**
NREM N1–N2	~	√	√	~	~	~	~	~	x
NREM N3 (SWS)	~	√	√	~	√	~	~	~	x
REM	x	√	√	~	x	~	~	~	~

*Ticks (√) indicate behavioral states required to be in the sleep state. Crosses (x) indicate behavioral markers that cannot occur during the sleep state. Dashes (~) indicate behavioral states that may or may not occur during the sleep state (36, 59, 72, 73)*.

**Figure 3:**
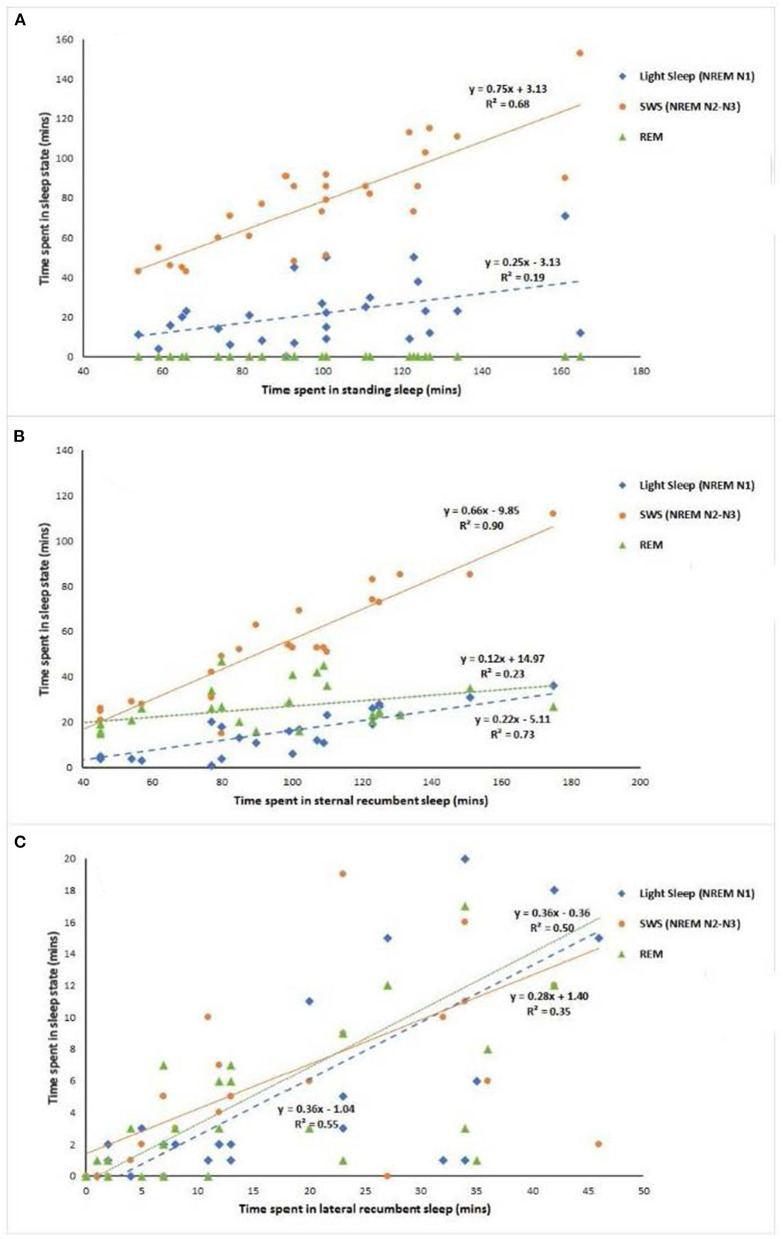
Linear regression analysis of average duration EEG sleep states against average duration behavioral states for 7 horses over 4 nights. Data taken from Kalus (42) [**(A)** standing, **(B)** sternal recumbency, **(C)** lateral recumbency].

Individual horses thus appear to have different strategies of sleep with different proportions of sleep states occurring in different behavioral positions. For example, in an EEG study of seven horses (42), the quantity of total sleep in the standing position ranged from 26.4% in one horse to 65.7% in another, in sternal recumbency it ranged from 21 to 59.7% and in lateral recumbency from 1.8 to 13.9%. By comparing EEG data with behavioral data, it may be possible to derive better estimates of sleep state from the behavioral assessment of the animal. For example, by plotting the duration of the EEG sleep state against the duration of associated behavioral state, the linear regression equation that is derived from this plot can be used to more accurately estimate the sleep state from the behavior of the animal. To illustrate this, Figure 3 presents the linear regression of the average duration of EEG sleep states (Light sleep, N1; SWS N2–N3; REM) for 7 horses (over 4 nights) against the average duration of sleep behavioral states (standing sleep; sternal recumbent sleep; lateral recumbent sleep) [data taken from Kalus (42)]. From Figure 3A, it can be seen that both light sleep (NREM N1) and SWS sleep (NREM N2–N3) can occur whilst the horse is in standing sleep with predominantly more time spent in the latter sleep state compared to the former. The linear equations give the estimation of the time spent in each sleep state when the animal is in standing sleep (Light sleep [NREM N1] = 0.25 × Total time spent in standing sleep – 3.13; SWS [NREM N2-N3] = 0.75 × Total time spent in standing sleep + 3.13). Interestingly, the variation within this estimation (as indicated by the R^2^ value) can be reduced by taking into consideration other behavioral features during the sleep period. For example, the number of wake sequences (periods of wakefulness during sleep cycles that are less than 3 mins) that occur within the total period of sleep increases the accuracy of the estimation. Taking the Kalus (42) data for example, including the wake sequence data into the regression analysis marginally increases the *R*^2^ value for Light sleep (NREM N1) from 0.19 to 0.34 and for SWS sleep (NREM N2–N3) from 0.68 to 0.74.

The two other primary behavioral sleep states within which sleep can occur are sternal and lateral recumbency. For sternal recumbency, all three sleep states are present as can be seen in Figure 3B. SWS (NREM N2–N3) predominates [SWS (NREM N2–N3) = 0.66 × Total time spent in sternal recumbency – 9.85] followed by REM sleep (REM= 0.12 × Total time spent in sternal recumbency + 14.97) and then light sleep (NREM N1) [Light sleep (NREM N1) =0.22 × Total time spent in sternal recumbency – 5.11]. Again, adding in additional behaviors into the estimation analysis can increase the *R*^2^ value and the accuracy of the estimation. In this instance, the inclusion of the total number of wake sequences into the regression analysis marginally reduces the variation of estimation from *R*^2^ value for Light sleep (NREM N1) from 0.73 to 0.74, from 0.90 to 0.91 for SWS sleep (NREM N2–N3) and from 0.23 to 0.24 for REM sleep. For lateral recumbency, again all three states can occur as illustrated in Figure 3C, but with a more equal distribution across the three states [Light sleep (NREM N1) =0.36 × Total time spent in lateral recumbency – 1.04; SWS (NREM N2–N3) = 0.28 × Total time spent in lateral recumbency + 1.24; REM= 0.36 × Total time spent in lateral recumbency – 0.36]. Again, inclusion of the total number of wake sequences into the regression analysis marginally reduces the variation of estimation from *R*^2^ value for Light sleep (NREM N1) from 0.55 to 0.57, from 0.35 to 0.36 for SWS sleep (NREM N2–N3) and from 0.50 to 0.51 for REM sleep. Further EEG studies that monitor in close detail the changes in behavior of the horse as it transitions between the three primary sleep states has the potential to increase the accuracy of behavioral sleep analysis through this multiple regression approach.

## Measuring Sleep Quality

Whilst sleep quantity and quality are inextricably linked, they are also often dissociated and thus it is important to take separate measures of both particularly in the context of “sleep deprivation” and animal welfare (74). In this context, it is important to have a definition of optimal sleep quantity and quality for any given species. It is also important to identify factors that can affect sleep quality and quantity that may produce a state of sleep deprivation in the animal. In this section, we will discuss the concept of sleep quality and how it can potentially be measured in the horse.

### General Concepts of Sleep Quality vs. Quantity

Although the average (and thus potentially optimal) quantity of total sleep time for a range of animal species is well documented (3), sleep quality is an uncommon measurement within animal sleep research and is therefore very poorly defined for the majority of mammalian species. In humans, the subjective experience of sleep quality has been quantified using sleep continuity measures such as reduced latency to sleep onset, the number of awakenings and duration of wakefulness after sleep onset (75) that often correlate with a reduction in total sleep time. Poor human sleep quality is also associated with patterns of sleep fragmentation or interruptions described as sleep that is punctuated by repeated periods of waking throughout the night (76). Fragmentation can involve transient arousals [transition to brain alpha activity (2 s or more)] and body movements lasting 0.5 s or longer (68), or wake sequences (<3 mins) (19) that are not associated with a change of sleep stage. Reduction in both sleep quality and quantity produce a state of sleep deprivation and subsequent sleep debt (77). Sleep deprivation is defined as either a complete lack of sleep or a shorter than optimal sleep time (74), for example, in humans this is quantified as <6 h of sleep per night (78). A distinction is also made between acute and chronic sleep deprivation based on the number of days the individual experiences less than the optimal sleep time. For example, in humans acute sleep deprivation has been defined as three consecutive nights of restricted or no sleep (79) whereas chronic deprivation has been described as persisting over longer periods of time e.g., 14 consecutive nights of restricted sleep (80). In animals, the former has been associated with reduced energy whereas the latter has been associated with generalized inflammatory and stress responses in the brain (81) leading to the death of the animal (82).

Sleep deprivation normally leads to recuperative or recovery sleep (80, 83). For example, human individuals maintained on a sleep wake pattern that induced a reduction in total sleep time were described as sleepier/less alert (77). The effects were reversed *via* extended sleep post sleep reduction, and the individuals were described as having “repaid the sleep debt” (77). The sleep debt can also be repaid by higher intensity sleep in the form of deeper slow wave sleep, where EEG slow wave activity (SWA) observed during NREM sleep is considered to represent a parameter of sleep intensity (9). In this context, slow wave sleep has also been described as a function of the duration of prior wakefulness (84) where it occurs closer to the point of sleep onset during the sleep cycle (85), therefore providing an efficient mechanism by which to recover the sleep debt if required. After sleep deprivation, increased levels of SWA during NREM sleep are also associated with a decreased number of spontaneous awakenings and an increased threshold for induced awakening (84, 86), which are characteristic of deeper/more intense/higher quality sleep. REM sleep is less sensitive to sleep deprivation, however, sustained deprivation of REM sleep will result in subsequent elevated levels of REM sleep that are not always immediate (as compared to SWS) but last over several nights (9). To summarize, whilst acute changes to sleep cause an immediate, short-lasting compensatory SWS response, only a severe deficit in REM sleep results in a rebound which is often delayed and prolonged (9).

### Potential Measures of Sleep Quality in the Horse

Recent human research (87) has demonstrated dysfunctional inflammatory responses during fragmented sleep which may therefore act as a biomarker for poor sleep quality. Salivary cortisol concentrations also change during awakening and subjective reports of poor quality sleep in humans and thus may also provide a useful marker of sleep quality (88). Behavioral studies also have the potential to increase the resolution of sleep data beyond total sleep time in order to provide a more detailed profile of the generalized equine sleep pattern and thus quality of sleep (Table 3). For example, the average duration of NREM sequences (total time of consecutive NREM sequences not interrupted by REM or wakefulness) (ranging from 3.37 to 9 min) and the number of these sequences (31.96 [18–52]) within the sleep profile may provide an indirect measure of the quality of sleep experienced by the animal. Similarly, the average duration (ranging from 0.91 to 5.22 min) and number of REM sequences (7.11–14.6) in the horse may act as an important indicator for sleep quality, as has previously been demonstrated in humans (89). However, it should also be noted that whilst abnormally high levels of REM sleep might be indicative of prior REM sleep deprivation, it can also act as a marker of stress and depression and thus it is important to establish the range of normal baseline values for any given species (90).

Additional measures of the equine sleep profile that may also be useful in quantifying equine sleep quality are the total duration of NREM sleep and also the number of wake sequences (<3 min) or micro-arousals within a sleep cycle. Previous work in humans has shown that an increased number of wake sequences negatively correlate with subjective sleep quality, whilst increased quantity of NREM sleep positively correlates with better motor function and accuracy (89). As indicative baseline values for horses, the mean total duration of NREM sleep is 178.74 mins and the mean number of wake sequences within a sleep cycle is 6.89 (Table 3).

A potential approach to transforming these measurements of equine sleep into a metric of sleep quality, is to develop an equine sleep quality index (SQI). Using the data from Table 3 and additional data from Kalus (42), one example is given below that uses some of the sleep duration parameters discussed as well as the number of wake sequences. Incorporation of additional sleep parameters (e.g., average number/duration of NREM sequences, EEG micro-arousal events) may further increase the resolution of the index and form the basis for future research. Consideration may also be given to different weightings for the different elements of the index as this research develops.


$$\begin{array}{l} \text{SQI = Total NREM duration/no. of NREM wake sequences }\\ \text{ + Total REM duration/no. of REM wake sequences.} \end{array}$$

Using Kalus (42) data as example:


$$\begin{array}{l} \text{SQI}=178.74/1.98+40.3/4.91=98.48 \end{array}$$

In summary, many equine studies fail to report in detail the nuances of the sleep cycle, with TST (NREM and REM duration combined) predominating as the primary measure of sleep quality. These data are undoubtedly useful to understand if a horse is sleeping and the impact of the environment on sleep, however, measuring the frequency and duration of wake and sleep state sequences may provide better metrics of sleep quality relative to sleep deprivation. These measures have the potential to form the basis of an equine sleep quality index, that can be compiled using both behavioral and/or EEG data.

## The Interrelationship Between Reduced Welfare/Exposure to Stress and Reduced Animal Sleep

**Figure 4:**
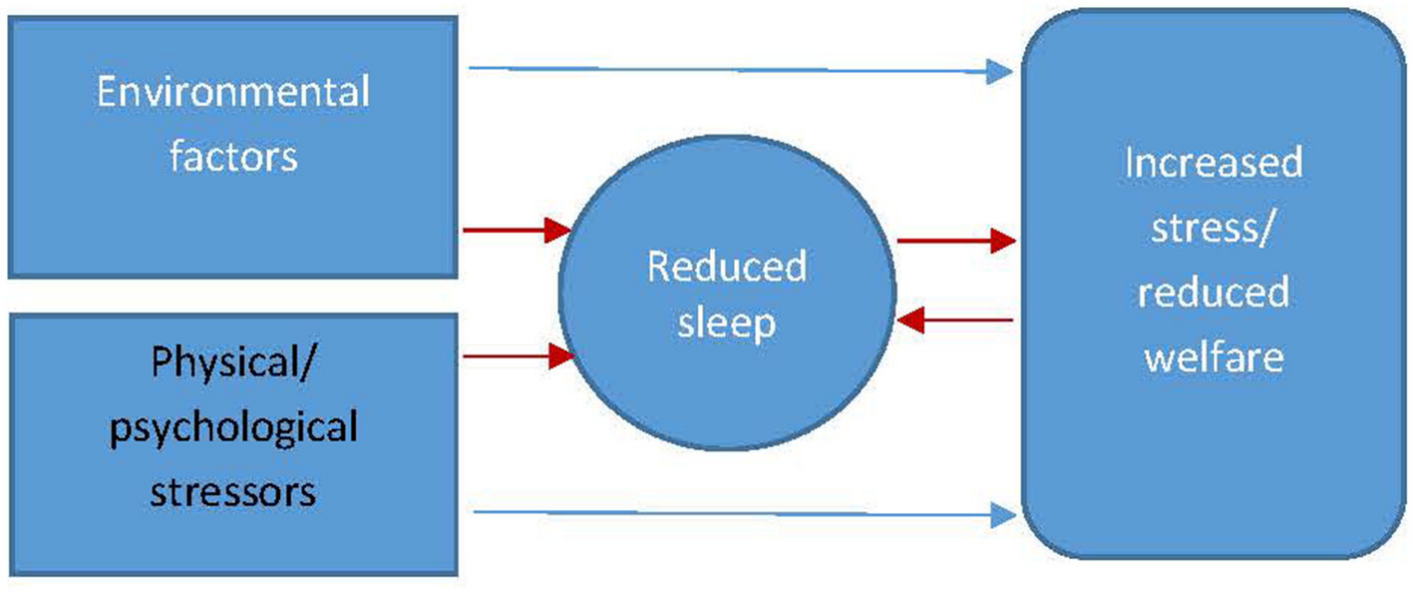
The interrelationship between factors of wellbeing affecting sleep and sleep affecting wellbeing (red arrows indicate the pathways to reduced sleep and the interplay between increased stress/reduced welfare and reduced sleep).

The central premise in the relationship between sleep and welfare (Figure 4) is that sleep deprivation has the potential to diminish animal welfare but also that factors affecting welfare (e.g., physical and psychological stressors, changes in environment) have the potential to affect sleep and thus further affect the welfare of the animal. In this section, we will discuss the interplay of these factors to gain a better understanding of the relationship between sleep quality and the welfare of the animal.

### Using Changes in Sleep as a Marker for Poor Welfare

It is often difficult to establish whether changes in sleep can be used as a marker for stress or whether changes in sleep are partially or wholly responsible for the animal being stressed. It is likely, as intimated in Figure 4, that changes in sleep are both a marker and cause of stress with the importance of the latter increasing over time as the quality of the animal's sleep progressively diminishes. For example in rodents, sleep has been used as a behavioral marker of stress alongside other standard biomarkers (adrenal weight, corticosterone) in response to cage size and social stress (91). It is difficult however to ascertain within this study whether it was social stress directly that was having the stress effect or whether the physiological response was due to the significant reduction in sleep duration. In this sense, reduced quality sleep may be a reasonable marker of stress in the first instance but ultimately becomes a compounding stressor in its own right over the longer term. When investigating the relationship between sleep and welfare, non-significant relationships have been reported between total sleep time, judgement bias, and behavior-based measures of welfare for shelter dogs (92) potentially highlighting the need for more sensitive measurements of sleep beyond total sleep time. As previously discussed, measurements of sleep quality such as number of disturbances or micro-arousals might yield more valid results. In the horse, very little research has been carried out on how welfare-reducing factors might manifest as changes in the equine sleep profile. One study reported that stereotypy horses spent significantly less time (*p* < 0.001) in REM (2.2 ± 1.7 vs. 6.7 ± 1.9) and N2–N3 SWS (13.8 ± 8.2 vs. 29.5 ± 3.4) sleep states and significantly more time (*p* < 0.001) spent in light sleep (N1) (22.6 ± 4.5 vs. 8.8 ± 3.4) (93) compared to control animals. Stereotypy is associated with current and/or historic states of reduced welfare (94) thus the result of this study, may suggest that reduced sleep is linked to reduced welfare in the horse.

### Effect of Sleep Deprivation on Animal Behavior and Welfare

Sleep deprivation and disorders in humans are well documented as constituting a major risk factor for psychiatric, cardiovascular, metabolic or hormonal co-morbidity and mortality (95). Sleep deprivation in humans has also been described as an anxiogenic factor with major impacts on the individual's welfare state (96). Sleep deprivation in animals reportedly causes serious physiologic changes including a state of high caloric ingestion without weight gain, reduction in anabolic hormones, opportunistic infections, and in some cases death (97). For example, sleep deprivation in rats has been shown to enhance bacterial infection as a result of immunosuppression (98). In human studies, sleep deprivation has been shown to produce hyperalgesic changes in healthy subjects, specifically SWS disruption due to its effect on the descending pain inhibitory control system measured through pressure pain sensitivity (99). Experimental animal studies have also reported hyperalgesia effects of REM or TST deprivation which appeared to prevent the analgesic action of endogenous and exogenous opioids (99). In terms of the effects of sleep deprivation on the welfare of the horse, much less specific research has been carried out. Excessive daytime sleepiness is known to increase risk of injury whilst cases of spontaneous equine collapse linked to sleep deprivation have been observed (100). Theoretically, horses will be susceptible to many of the clinical sequalae of sleep deprivation that has been observed in other species (Table 5). Further research is needed to establish whether these conditions are apparent in horses and whether they are associated with changes in the sleep profile of the animal.

**Table 5:** The after effects of sleep deprivation in human and rodent models.

**Sequalae**	**Species**	**References**
Impaired visual perception	Human	(101)
Reduced capacity to engage in tasks requiring simple sustained concentration/attention	Human	(102)
Impaired decision making including more high-risk strategies and reduced concern for negative consequences of these	Human	(103)
Impaired memory consolidation	Rodent	(104)
Negative effects on vigilance and simple reaction time	Human	(105)
Increased daytime sleep propensity/micro-episodes of sleep leading to lower capabilities and efficiency of task performance and to increased number of errors	Human	(68)
Poor memorization and schematic thinking,	Human	(106)
which yields wrong decisions	Rodent	(107)
Emotional disturbances such as deteriorated interpersonal responses and increased aggressiveness	Human	For review see Fairholme and Manber (108)
Changes to pain perception, specifically hyperalgesia	Rodent	For review see Lautenbacher et al. (99)

## Factors Reducing Sleep

In this section we discuss the primary factors affecting sleep quality and quantity generally and also specifically in the domestic horse as well as identifying clinical and non-clinical conditions for which reduced sleep may be symptomatic.

### Stressors

Both physical and psychological stressors can lead to a reduction in sleep duration and quality. Pain is an example of a physical stressor that influences sleep. For example, in a meta-analysis of human studies (109) using polysomnography (PSG) to quantify sleep in people with chronic pain (CP), 44% of those with CP were also diagnosed with a sleep disorder, most commonly insomnia, which was comparatively higher than the general population. The review also reported that, in terms of sleep architecture, people with chronic pain appear to spend more time in NREM N1 and experience greater sleep fragmentation than healthy controls. Mechanisms underpinning the relationship between pain and sleep disruption include the physical discomfort of pain and associations between CP and sleep disruption in a variety of brain-based changes such as alterations to the inflammatory response which is critical for sleep-wake regulation (109). Indeed in humans, chronic pain is described as comorbid with sleep disruption, recognizing that pain can be both the cause and consequence of altered sleep patterns (110) as it can reduce pain thresholds thus further enhancing the influence of pain (111)^.^ In large animals, conditions that induce pain such as arthritis are suggested to prevent the animal from adopting a recumbent position, resulting in reduced sleep and sleep disruption (112, 113). For example, chronic joint disease preventing recumbency has been associated with spontaneous collapse for captive elephants (114) and abdominal pain was associated with reluctance to adopt a recumbent posture in an equine case study (115). Geriatric horses may not choose to be sedentary due to secondary foot pain associated with excessive standing, further amplified by the pain in large mammals that comes with greater body mass. However little evidence exists to describe this or the influence of pain on the occurrence of equine sleep specifically.

Chronic pain, as a stress state, is one of the critical factors associated with depression in humans, and the coexistence of these disorders tends to further aggravate severity of both for the patient (116). Some human sleep disturbances (insomnia or hypersomnia) have been linked to states of depression (117), often compounded by the experience that all efforts to initiate sleep are unsuccessful leading to “learned helplessness” and a further state of depression (118). Depressive patients have been reported to exhibit reductions in sleep efficiency, shorter REM sleep periods (and latency), and increases in the number of awakenings (119). In animals, there is evidence that sleep deprivation contributes to the development of depression or anxiety-like symptoms and produces states of physiological stress (120–122) housing, (123). Horses in their usual domestic environments have been observed to perform unusual gaze, head and ear fixity, and indifference to environmental (tactile and visual) stimuli, which were likened to symptoms of “depressive syndrome” (124). This atypical posture differs to “standing rest” where comparatively the horse's neck is rounder and the eyes are at least partly closed (125). Little is known about the relationship between equine models of depression and sleep patterns, however horses displaying established stereotypic behavior are reported to display different nocturnal activity profiles (126). Recumbent behaviors of stereotypic horses are also reduced compared to non-stereotypic horses (126, 127), suggestive of sleep deprioritization or differing sleep strategies compared to non-stereotypic animals.

Beyond comfort, perceived safety within the environment also influences sleep. For example, human sleep is sensitive to a novel environment and stimuli, described by the “first night effect” (FNE) which has also been observed in dogs (128). Humans, however, have the capacity in most instances to modify the stress-inducing factors within the environment which is often in stark contrast to domesticated species that lack a level of control over factors within their environment. For example, moving horses from a period of turnout to overnight stabling has been shown to affect their daytime behavioral profile (129) whilst nocturnal recumbency significantly increased for the 6 weeks after horses were brought into an overnight stabling management regime from a period of overnight turnout (130). A study using EEG data suggested that horses require a period of acclimatization to novel environments (65, 131), observed as greater levels of vigilance displayed when horses are initially stabled after a period of turnout (129, 130). These studies suggest that stabling in isolation removes the aspect of shared safety through group vigilance during turnout, whilst presenting a wealth of novel (auditory and other) sensory stimuli all of which have an effect of the equine sleep profile.

In animals, the psychological aspect of stress becomes an important factor relative to its effect on sleep. For example, in rats and mice, the occurrence of sleep after stress appears to be highly influenced by situational variables including whether the stressor was controllable and/or predictable, whether the individual had the possibility to learn and adapt, and by the relative resilience and vulnerability of the individual experiencing stress (132). In this respect, deeper or longer NREM sleep reportedly follows acute social stress (133, 134), whilst stress experienced in response to restraint is followed by a selective increase in REM sleep (86, 135). Similar findings have been reported in canine studies where stress-inducing experiences (e.g., short periods of isolation on a leash followed by approach by a stranger) resulted in increased sleep (136). The increase in sleep states reported in animals post-stress contrasts with sleep reductions often observed in humans, where stress-based memories of past events as well as worries and expectations can disrupt and reduce human sleep. In that respect, compared to some animals, the human brain has the capacity to turn a single acute stressor or previous life event, or even one situated in the future, into a persistent and chronic stress state (132). Other psychological stressors in humans have also been reported to reduce the quality of sleep through increased levels of sleep fragmentation (137). Sleep deprivation can also further sensitize the individual to stressful stimuli and events (138) thus further compounding the problem. As previously discussed in horses, changing the animal's sleep environment from pasture-kept groups to single housed stabling has been reported to significantly reduce total sleep time (130). This suggests that the psychological stressor of changing the social environment can have a significant impact on sleep in the horse. Again, further work identifying the exact aspects of psychological stress in the horse that affect equine sleep is needed.

### Environment

Light is one of the most important environmental factors affecting sleep across a range of species. The sleep-wake cycle is driven by a central clock, the superchiasmatic nucleus (SCN), and in most mammals, by changing concentrations of melatonin due to light exposure (139). Photoentrainment of sleep to circadian rhythms is usually mediated by photoreceptors that detect changes in the quantity and quality of light over the 24 h dawn/dusk cycle (140). The sleep-wake cycle is vulnerable to changes in the timing of circadian rhythms (phase shifting) (141) *via* exposure to bright light at specific points during the light-dark cycle, even during sleep. Because of the increased use of artificial light within society, humans tend to spend less time in the dark which has been described as influential in the shift from biphasic to monophasic sleep patterns (142). This includes exposure to artificial light at night (ALAN), which could be considered an environmental stressor due to the fact that it has been shown to disrupt the biological clock *via* suppression of melatonin (143). Prolonged exposure to ALAN induces adverse effects on mood and productivity (144), and in laboratory rodents has been associated with reduced anxiety-related behavior including more time spent in the open (145). The latter is considered a maladaptive response specifically for urban dwelling prey species (145). Changes in exposure to light and associated phase shifts (waking earlier or later) are important to humans, enabling them to adjust to travel across time zones or facilitating adaptation to night shift work or early awakening (146). However, sleep disruption can be a biproduct of these phase shifts, for example in the form of “jet lag” (147). Domesticated horses are often housed in situations that include artificial light, although the way in which this acts as a zeitgeber for equine sleep is little understood. A recent study specifically examined the effect of overnight light on sleep behavior in horses and reported a significant reduction in sternal recumbency linked with the REM sleep state (57). Interestingly breeding mares are routinely exposed to artificial light to manipulate the breeding cycle in the northern hemisphere horse racing industry (148) but very little is known about the impact of this procedure on the quality of sleep for those animals. The use of red light at night has been advocated for use within equine husbandry as a means to minimize circadian disruption (149). Competition horses are regularly traveled internationally although little is known about the effects of changing time zones and rates of adaptation relative to sleep and performance.

Non-photic zeitgebers for sleep include physical and social activity. Experimental studies on the effects of exercise for human sleep patterns have described increase TST, prolonged REM latency, decreasing REM sleep and increasing SWS sleep (150). Physically active individuals also report less daytime tiredness, better subjective sleep and fewer sleep problems than sedentary individuals (151, 152). The effects of physical exercise on sleep are known to depend upon the time the exercise is performed (153), fitness and the intensity of the exercise (154), and other exogenous and endogenous factors linked to the general wellbeing of the participant (155). Daily routines and social rhythms are also linked to good human sleep, for example, self-reported good sleepers have more daily activities, earlier daily scheduling of their social rhythms, social rhythms characterized by greater regularity, and are involved in more activities with active social engagement than poor sleepers (156). Overall, exercise has been described as a robust zeitgeber of sleep acting *via* skeletal muscle clocks (157) that have an important role in regulating the mammalian circadian system generally (158). In horses, groups of animals will demonstrate both rest and locomotory synchrony (159, 160) and this can be significantly affected by stabling and social conditions. For example, horses at pasture demonstrate synchronized ultradian rhythmicity in patterns of locomotion that are much weaker when the horses are stabled (160). This strongly suggests that, for the horse, there is a state of endogenous circadian periodicity that acts irrespective of light and social cues (160). Research investigating the circadian 24-h expression of exercise relevant genes in equine skeletal muscle has concluded that metabolic muscle capacity is influenced by scheduled exercise, with significant interactions between circadian time and exercise for specific muscle genes (161). On the basis of these results, it has been suggested that optimal performance may be achieved when competition and scheduled training times coincide (161). Little is known, however, about how this might result in phase shifts for sleeping and further investigation is necessary to understand how this and overlying social factors affect the occurrence of equine sleep.

Other non-photic entrainment factors/zeitgebers for sleep include temperature and humidity. Sleep and rest in many mammalian species are associated with a reduction in core body temperature (CBT) (162), a thermoregulatory process whereby heat is redistributed from the core to the outer layer of the body. The CBT rhythm is suggested to be able to entrain peripheral pacemakers around the body and can affect normal sleep patterns. For example, increasing distal skin blood temperature *via* exercise (163) is considered to be one of the factors that can reduce the latency of sleep onset (164). Sleep is also highly susceptible to environmental heat, as demonstrated in rats (165), and in cows heat stress is known to reduce lying time that can subsequently impact on sleep levels (166). Meanwhile, recent work by Yadhapalli et al. (167) suggests that sleep-promoting circadian clock neurons are inhibited by heating and excited by cooling in *Drosophila melanogaster* with evidence that these neurons are continuously integrating temperature changes to coordinate the timing of sleep and activity. Horses tend to sleep less during higher (58) and longer during lower (168) ambient temperature when theoretically core heat redistribution is harder and easier, respectively. However, this also tends to coincide with changes in daylight season and thus it is difficult to identify the predominating sleep-affecting factor. According to Duncan (169), free-living Camargue horses adopt recumbent positions more so in spring with a higher prevalence of standing alert and walking during the summer although these behavioral patterns are also thought to be driven by availability of forage (specifically crude protein) and the presence of biting flies. During the autumn and winter months, these horses also increased the proportion of time spent resting whilst standing but with decreased time spent in a recumbent posture, correlating with low ambient temperatures and increased rain (169). All in all, equine sleep-related behavioral patterns appear closely associated with environmental seasonal fluctuations with temperature being an important factor in this respect. However, limited research exists specifically on the direct effects of ambient temperature on equine sleep and CBT, which maybe important given that many horses have their coats clipped, are fitted with rugs and experience different climates due to international travel.

Non-zeitgeber environmental factors affecting sleep also pertain to whether the environment is safe and comfortable and facilitates species-specific sleep postures. Humans commonly sleep in preferred sleep sites (e.g., bedrooms) on surfaces designed to provide support and comfort during recumbency (e.g., mattresses) and these sites/surfaces may be shared. Many aspects of sleep quality can be affected by perceived (dis)comfort of the sleeping surface [e.g., (170)], but also by the presence of co-sleepers [e.g., (171)]. In addition, the relative merits of different human sleep postures (prone, surpine and lateral) are also discussed relative to sleep quality [e.g., (172)]. Some animal species sleep only at specific sites (173), others may utilize multiple sites with specific characteristics. In non-human primates, Anderson (174) identified influential factors associated with comfort and the selection of sleep sites, these included thermal comfort, noise reduction and postural demands during sleep. In cows, management factors such bedding type (175) and stall design (176), can significantly influence lying time which is known to impact on the sleep quantity (177). The domesticated horse often has access to pasture at night offering a larger area from which to select sleeping sites although little is known about preferred sites and their influence on sleep quantity and quality. Anecdotally, horses that are stabled overnight are reported to display recumbency in a preferred area of the stable, which is often different to standing sleep sites. Significantly longer bouts of recumbency have been reported for horses kept in stables with larger surface areas, suggesting that this factor influences motivation to adopt recumbent positions (178, 179). In this respect, a larger surface area might facilitate maneuverability which is essential to achieve recumbent positioning and critically important to enable the horse to effectively achieve REM sleep. Within the stable, the characteristics of the sleep surface have also been shown to influence sleep-related behavior. For example, straw as a bedding substrate is consistently associated with higher proportions of recumbency as part of the nocturnal time budget when compared to wood shavings (61, 180, 181), and other bedding substrates such as peat moss/shavings mix and crushed wood pellet (63). The depth of the bedding substrate used within the stable is also noted to have a significant effect on nocturnal behavior, where lower depths (<10 cm) of bedding appear to significantly reduce the occurrence of recumbent behavior regardless of bedding substrate (57, 58).

The level of stimulation from the environment may also influence sleep due to varying levels of arousal and alertness. In humans, an example of a hyper-stimulating environment would be an Intensive Care Unit (ICU), with evidence of very poor sleep occurring during stays in ICU due to noise, critical illness itself, and treatment events throughout the day and night (182). During sleep, noise is generally considered to be an unwanted auditory stimulus (183) whereby the human auditory system continues to scan, evaluate and react to environmental sounds even whilst asleep where more meaningful noise events are more likely to cause arousals from sleep than those events that are not (184). The depth of the sleep phase, background noise level and individual characteristics affecting sensitivity to noise are known to determine whether or not noise will disturb sleep (185–187). Other factors include the type of noise (e.g., continuous, intermittent, impulsive), noise intensity, noise frequency, noise spectrum, and noise interval (e.g., duration, regularity, expected) (183). It is currently unclear how many additional noise-induced awakenings are acceptable/without consequences for sleep recuperation and health, especially given the large inter-individual differences in susceptibility to noise. Prey species typically tend to remain vigilant for the rest of the night, even after initial adaptation to the nocturnal environment, following one awakening elicited by a spontaneous/startling stimulus (3). However, continuous auditory stimulation provided overnight (e.g., music) can have a masking and relaxing effect in animals (62, 188). For example, in horses, overnight music appeared to facilitate increased displays of biologically significant behaviors including lateral recumbency and the behavioral benefits continued beyond the enrichment period (62).

Conversely, hypo or low levels of stimulation can also have a dramatic impact on sleep. Low levels of stimulation (often connotated with boredom) (189) leads to lethargy and mental fatigue that may result in the animal sleeping earlier than usual or resting more, as the environment offers no opportunities to keep them awake or tire them out (189, 190). In this sense, increased TST is not always an indicator of positive welfare. Several studies (50, 151, 191) have shown that sleep quality is related to daily activity level, such that poor sleep quality arises from inactivity or proneness toward sedentary lifestyles. Horses displaying depressive-like forms of waking inactivity may be mistakenly observed as standing at rest or standing asleep due to the general similarities in the behavioral ethogram (124). However, they may in fact not be achieving species-specific optimal sleep due to the hypo-stimulating environment.

In summary, whilst most horse management systems seek to provide optimal husbandry conditions, the domestic stable environment potentially creates a number of challenges from a sleep quantity/quality perspective. Whilst some research has been carried out on the impact of some of these factors (e.g., bedding and light) and how sleep can be improved in the stable environment (e.g., music), much more research is needed to further investigate these and other factors (e.g., exercise, social contact, changing environments and perceived threat) as well as sleep outside of the stable environment (e.g., at pasture). The impact of regular environmental changes for competition horses, traveling nationally and internationally, also needs due consideration.

## Discussion and Future Directions

Understanding the evolutionary function of sleep has been widely regarded as one of the greatest challenges for ethological research. Researchers have identified variation in sleep duration in a range of species, with some suggested factors linked to the major forces driving the occurrence of sleep, including risk of predation (the sleep exposure index), gestation period and neonatal body mass, body mass, encephalization, and basal metabolic rate. One of the primary aims of this review was to establish, through a review of the literature, a detailed profile of normal equine sleep. We provided a summary table of all equine studies to date to establish both normal sleep quality and quantity that will be a useful reference tool for establishing baseline levels of quantitative and qualitative metrics of horse sleep. The table, however, also highlighted that the majority of studies commonly reported total sleep time and that there were a limited number of studies that measured (a) the different stages of sleep and (b) sleep across the 24 h period. We recommend that future studies should focus on determining what “normal” equine sleep is, through 24 h sleep profiles that describe the duration and frequency of NREM/ REM cycles as well as sequences of wakefulness. This will yield novel information about the equine sleep profile and provide a deeper understanding of equine sleep quantity and quality. In addition, to better understand levels of variation between horses, more research is needed into the effects of standard variables of age, sex and breed on the different measurements of equine sleep.

Technologically, there is now an opportunity to improve the accuracy of sleep quantification in animals through mobile wireless EEG and polysomnography (PSG) equipment. This will also greatly increase the level of equine EEG sleep data that, to date, has come from a limited number of sources over limited observation periods with a lack of precision measurements of sleep quality (e.g., NREM/REM cycles, wake sequences). Further EEG studies that monitor in close detail the changes in behavior of the horse as it transitions between the three primary sleep states also has the potential to increase the accuracy of behavioral sleep analysis. In this review, we demonstrated that EEG data can be used to refine the behavioral analysis of sleep through a multiple regression approach. Further EEG studies with simultaneous detailed behavioral monitoring of equine sleep will further refine this multiple regression methodology. Moreover, the inclusion of automated measures of behavior (e.g., movement data loggers, vision motion analysis) alongside EEG sleep data has the potential to automate animal sleep scoring with high levels of accuracy. In the meantime, although behavioral measurements of sleep lack the precision of EEG or PSG, these measurements are easily accessible alternatives that can achieve valid measurements of sleep including sleep fragmentation. The review also provided a strong rationale for developing an equine sleep quality index, with a particular emphasis on assessing wake sequences/sleep fragmentation, in order to better assess factors affecting sleep in the horse.

During the review, primary factors affecting sleep were explored under the categories of physical (pain) stressors, psychological (perceived safety, social isolation, hypo-stimulation) stressors, and aspects of the environment (light, bedding substrate, physical and social activity, noise and temperature and humidity). It became clear that whilst there was a strong relationship between environmental stressors, sleep and welfare, it was not always clear as to the direction of the relationship. For example, whilst reduced sleep quantity/quality may initially be a marker of stress, it can also become a compounding stressor in its own right over the longer term. In this respect, much more research is required to disentangle the relationship of reduced sleep as a marker of stress vs. reduced sleep acting as a stressor. Additional sleep-affecting factors that still need to be investigated in the horse include emotional state, social environment, the influence of light on circadian control of sleep, levels of exercise and nutritional factors. There are also unanswered questions in relation to training and competition schedules, for example, do regular exercise schedules help promote sleep and is there an optimal time to exercise relative to optimizing sleep? Furthermore, do animals under intense training schedules sleep more than when they are not, and how might we facilitate that relationship? Little is also understood about how core body temperature acts as a cue for sleep and rest patterns, especially in comparison to social rhythms of group housing or turnout. For example, is it possible that clipping and rugging horses could result in a phase shift in sleeping patterns? Again, these questions provide a huge opportunity to extend the currently limited field of equine sleep research.

In addition to assessing factors that affect equine sleep, compensatory mechanisms, that exist for short term sleep reductions in a range of animal species, is not well defined in the horse. It is also not known at what point sleep deprivation becomes chronic and how this impacts the welfare of the horse particularly in the context of spontaneous collapse. Research has also identified that reduced sleep quantity and quality affects cognitive (e.g., memory) function and motor performance in a range of animal species but very limited research has been carried out in this area in the horse. Again, further research into these areas will help identify the levels of sleep disturbance that the horse can tolerate from both a performance and welfare perspective.

## Author Contributions

LG and SM: equal contribution to concept and writing. LG: editing. Both authors contributed to the article and approved the submitted version.

## Conflict of Interest

The authors declare that the research was conducted in the absence of any commercial or financial relationships that could be construed as a potential conflict of interest.

## Publisher's Note

All claims expressed in this article are solely those of the authors and do not necessarily represent those of their affiliated organizations, or those of the publisher, the editors and the reviewers. Any product that may be evaluated in this article, or claim that may be made by its manufacturer, is not guaranteed or endorsed by the publisher.
